# New SDS-Based Polyelectrolyte Multicore Nanocarriers for Paclitaxel Delivery—Synthesis, Characterization, and Activity against Breast Cancer Cells

**DOI:** 10.3390/cells12162052

**Published:** 2023-08-11

**Authors:** Marzena Szwed, Sylwia Michlewska, Katarzyna Kania, Marta Szczęch, Agnieszka Marczak, Krzysztof Szczepanowicz

**Affiliations:** 1 Department of Medical Biophysics, Institute of Biophysics, Faculty of Biology and Environmental Protection, University of Lodz, Pomorska 141/143 St, 90-236 Lodz, Poland; agnieszka.marczak@biol.uni.lodz.pl; 2 Laboratory of Microscopic Imaging and Specialized Biological Techniques, Faculty of Biology and Environmental Protection, University of Lodz, Banacha 12/16 St, 90-237 Lodz, Poland; sylwia.michlewska@biol.uni.lodz.pl; 3 Laboratory of Virology, Institute for Medical Biology, Polish Academy of Sciences, Lodowa 106 St, 93-232 Lodz, Poland; kkania@cbm.pan.pl; 4 Jerzy Haber Institute of Catalysis and Surface Chemistry, Polish Academy of Sciences, Niezapominajek 8 St, 30-239 Kraków, Poland; marta.szczech@ikifp.edu.pl (M.S.); krzysztof.szczepanowicz@ikifp.edu.pl (K.S.)

**Keywords:** nanocapsules, taxanes, EPR effect, apoptosis

## Abstract

The low distribution of hydrophobic anticancer drugs in patients is one of the biggest limitations during conventional chemotherapy. SDS-based polyelectrolyte multicore nanocarriers (NCs) prepared according to the layer by layer (LbL) procedure can release paclitaxel (PTX), and selectively kill cancer cells. Our main objective was to verify the antitumor properties of PTX-loaded NCs and to examine whether the drug encapsulated in these NCs retained its cytotoxic properties. The cytotoxicity of the prepared nanosystems was tested on MCF-7 and MDA-MB-231 tumour cells and the non-cancerous HMEC-1 cell line in vitro. Confocal microscopy, spectrophotometry, spectrofluorimetry, flow cytometry, and RT PCR techniques were used to define the typical hallmarks of apoptosis. It was demonstrated that PTX encapsulated in the tested NCs exhibited similar cytotoxicity to the free drug, especially in the triple negative breast cancer model. Moreover, SDS/PLL/PTX and SDS/PLL/PGA/PTX significantly reduced DNA synthesis. In addition, PTX-loaded NCs triggered apoptosis and upregulated the transcription of Bax, AIF, cytochrome-c, and caspase-3 mRNA. Our data demonstrate that these novel polyelectrolyte multicore NCs coated with PLL or PLL/PGA are good candidates for delivering PTX. Our discoveries have prominent implications for the possible choice of newly synthesized, SDS-based polyelectrolyte multicore NCs in different anticancer therapeutic applications.

## 1. Introduction

Breast cancer is the second most common malignancy and the fifth most common cause of cancer death in women. In 2020, almost 2.3 million new breast tumour cases were diagnosed, and 690,000 women lost their lives worldwide [1]. The main course of action for pre-invasive and invasive breast cancer treatment includes surgery in combination with radiation therapy and systemic treatment, i.e., hormone therapy or chemotherapy [2]. According to the oncologist community, a combination of taxanes and anthracyclines is still considered as an effective tool in the fight against cancer [3]. However, the application of these chemotherapeutic drugs has three main limitations. First, a significant fraction of the drug molecules finishes up in normal tissues, causing severe side effects, and limiting the dosage of the therapeutic drug [4]. Second, there is a loss of therapeutic effectiveness over the course of chemotherapy, through obtained multidrug resistance (MDR). This phenomenon is found in up to 50–70% of breast cancer patients undergoing conventional chemotherapy [5]. Last but not least, the significant hydrophobicity of therapeutic substances is a big obstacle for the pharmaceutical industry that needs to be overcome [6].

In this context, new therapeutic tools are required and nanotherapeutic drugs should be explored as a promising approach to offer clinicians new possibilities to treat such diseases. Nanomedicine-based therapeutics have been developed to overcome the lack of non-controlled activity of clinically applied anticancer drugs [7]. One of the major aims of nanomedicine is the reformulation of well-established chemotherapeutic drugs that may be helpful in improving the delivery of anticancer agents into tumour tissue [8]. Subsequently, NP encapsulation may increase the solubility and stability of a drug, enabling increased circulation times and bioavailability. In addition, drug delivery systems can be designed to improve the specificity of distribution and bypass important physiological barriers [9]. Paclitaxel (PTX) is one of the most efficient chemotherapeutic agents and is mainly used over the course of treatment of lung, ovarian, and breast cancer [10]. PTX is mainly known as an antimitotic chemotherapeutic drug that suppresses the polymerization dynamics of microtubules, following the arresting of mitotic spindle formation due to activation of the mitotic checkpoint. This drug diminishes the critical number of tubulin molecules required for its assembly; therefore, arranging the extension of the tubulin polymer [11]. Although an activation of the spindle assembly checkpoint is the main mechanism of PTX, it has been shown that it also causes the activation of programmed cell death pathways [12]. However, the high efficiency of PTX as an anticancer agent is diminished by its association with several side effects, which include hypersensitivity reactions referring to damage to the nerves located outside of the brain and spinal cord, acute cardiomyopathy, and gastrointestinal dysfunction [13]. Therefore, the encapsulation of PTX within various nanocarriers (NCs) may protect drug molecules from accelerated exclusion from the body, maintain their anticancer toxicity, and increase pharmacokinetics [14,15].

As endothelial pores in angiogenic vessels vary in size from 10 to 1000 nm, passively targeted NCs (20–200 nm) can accumulate inside the tumour interstitial space. This phenomenon, known as the enhanced permeability and retention (EPR) effect [16], is supported by the poorly functional lymphatic vessels in tumours leading to inefficient drainage from the tumour extracellular space [17]. Nano-formulations of PTX with a diameter larger than 50 nm would result in better uptake in tumours due to the EPR effect. Following a lot of dedicated efforts during in vivo studies, clinical trials have been performed and nine types of PTX nano compounds such as Abraxane^®^, Lipusu^®^, Cynviloq^®^, and Nanoxel^®^ are clinically available these days [18]. On the other hand, the clinical application of nano-PTX has alerted more researchers that the therapeutic effectiveness of PTX encapsulated in various nanosystems is far from satisfactory. For instance, the limited accessibility of the drug released from the NCs and the required higher doses trigger serious toxicity, mostly due to unsatisfactory off-target distribution [19]. 

Among the numerous PTX nano-encapsulation procedures, the sequential adsorption of charged nanoobjects, also known as the layer-by-layer (LbL) method, has recently arose as one of the most investigated methods for the formulation of promising drug carriers [20,21,22]. The advantages of the formulation of nanoemulsion droplets as the core of nanocapsules synthesized by the LbL method include easy production, functionalization, biocompatibility, maintained drug release, and retarded degradation rate [23]. While the costly and energy-intensive filtration and centrifugation methods have been used for LbL-synthesized nanocapsules based on solid matrices, the saturation method is used for the construction of polymeric nanocapsules based on liquid cores [24]. Subsequently, during the LbL technique, surfactants may be used that, together with the oppositely charged polyelectrolytes, form a single core nanocapsule [25]. It has been previously shown that an in-house technology consisting of biodegradable nanoparticles (NPs) stabilized by the docusate sodium salt/poly-L-lysine surface complex (AOT/PLL) can be used to deliver taxanes, vitamins, and natural substances [26,27]. Keeping in mind the use of the final product as a drug carrier and the hydrophilic environment of the cancer cell, an alternative solution is to use water-soluble surfactants, which will form a multicore of polyelectrolyte NCs.

In the present study, a new method of synthesizing polyelectrolyte multicore NCs for hydrophobic drugs, based on the water-soluble surfactant sodium dodecyl sulphate (SDS), was developed, and the applicability of these NCs as drug delivery systems for PTX was also determined. Two forms of polyelectrolyte multicore NCs, one coated with poly-L-lysine (SDS/PLL) and the second coated with poly-L-glutamic acid—PGA (SDS/PLL/PGA) were tested. To verify the cytotoxicity of the examined nanosystems to in vitro cultures, two breast cancer cell models were used: MCF-7 and MDA-MB-231 cells, which have different sensitivities to oxidative stress and estrogenic receptor expression. Since multicore nanocapsules are dedicated to intravenous administration, it was crucial to evaluate a cytotoxicity of SDS/PLLL/PTX or SDS/PLL/PGA/ PTX towards human endothelial cells. Thus, in parallel, experiments were performed on a non-cancer human endothelial (HMEC-1) cell line The toxicity of the NCs was studied during a 96 h incubation with particular emphasis on ATP synthesis, the inhibition of metabolic activity, and a reduction in cell proliferation. Subsequently, the hallmarks of apoptosis related to disturbances in cell membrane asymmetry, caspase 3/7 activity, and mitochondrial stability were evaluated. Finally, an RT-qPCR was performed to check whether the expression of genes involved in mitochondrial homeostasis was altered by the PTX released from SDS/PLL and SDS/PLL/PGA NCs. 

## 2. Materials and Methods

### 2.1. Materials

Sodium dodecyl sulphate (SDS), poly-L-lysine hydrobromide (PLL, MW ∼15,000–30,000), poly-L-glutamic acid sodium salt (PGA, MW ∼15,000–50,000), 3-(4,5-dimethyl-2-thiazolyl)-2,5-diphenyl-2H-tetrazolium bromide (MTT), 5,5′,6,6′-tetrachloro-1,1′,3,3′-tetraethyl-benzimidazolcarbocyanine iodide (JC-1), chlorophenylhydrazone (FCCP), primers for RT-PCR (Table 1), trypsin-EDTA sodium chloride (NaCl), and phosphate buffered saline (PBS) were purchased from Sigma-Aldrich (Germany). Paclitaxel (PTX) was supplied by from Selleckchem, while absolute ethanol was from Avantor Performance Materials (Gliwice, Poland). Annexin V-FITC was obtained from BioVision Inc. (Milpitas, CA, USA). All materials were used without further purification. Other chemicals and solvents were of high analytical grade and were supplied by Sigma-Aldrich or Avantor Performance Materials Poland S.A. (Gliwice, Poland). The distilled water used for all solvents and buffer preparation was obtained with Millipore Direct-Q 5UV purification system. 

### 2.2. Preparation of Polyelectrolyte Multicore Nanocarriers

The polyelectrolyte multicore NCs were prepared (following the procedure explained in the Patent claim WIPO ST 10/C PL443843). Briefly, nanocores (emulsion droplets) were formed by addition of water-soluble surfactant SDS (10 CMC) to the PLL solution (200 ppm prepared in 0.015 NaCl) under gentle mixing with a magnetic stirrer. 

The optimal ratio of SDS to PLL was estimated by measuring the zeta potential of the obtained NCs and measuring the stability of their suspension with dynamic light scattering (DLS) measurements. Positively charged multicore polyelectrolyte (SDS/PLL) nanocarriers with a hydrodynamic diameter of approximately 80 nm were obtained. 

Furthermore, prepared multicore NCs were coated with PGA by adding multicore NCs SDS/PLL to PGA (2000 ppm) solution; again, the optimal ratio was examined by measuring the zeta potential of the obtained NCs (SDS/PLL/PGA) and examining their stability. Negatively charged SDS/PLL/PGA nanocarriers with a hydrodynamic diameter of approximately 105 nm were obtained.

To encapsulate the anticancer drug, PTX (61 mg/ml) was solubilized in SDS micelles with the addition of absolute ethanol (later evaporated) before the preparation of SDS/PLL NCs.

### 2.3. Determination of Particle Size, Shape, and Zeta Potential

The hydrodynamic diameter, size distribution, polydispersity index (PDI), and zeta potential were measured using a Zetasizer Nano ZS instrument (Malvern-Panalytical Ltd., Malvern, UK) by Dynamic Light Scattering (DLS) and micro-electrophoretic mobility, respectively. Each measurement was conducted in 0.015 M NaCl at 25 °C as an average of at least three runs with 10 measurements. Nanoparticle Tracking Analysis (NTA) was used to determine the concentration of the synthesized NCs with the NanoSight NS500 instrument (Malvern-Panalytical Ltd., UK). UV-vis absorption spectra were acquired to verify PTX encapsulation in the multicore NCs using a UV-1800 spectrophotometer (Shimadzu, Kyoto, Japan). In addition, transmission electron microscopy (TEM) was used to estimate the shape of SDS-based polyelectrolyte multicore NCs loaded with PTX. Ten microliters of each sample were vortexed, placed on 200 mesh copper grids with a carbon surface and incubated for 10 min. Subsequently, the samples were stained with uranyl acetate saturated solution for 20 min, washed in deionized water, and dried at room temperature. The TEM images were created using a JEM-1010 (Akishima-Tokio, Jeol, Japan).

### 2.4. Stability Studies

The stability of multicore NCs with time was assessed by visual observation at room temperature.

### 2.5. Cells and Treatments

MCF-7 (epithelial breast adenocarcinoma cell line) and MDA-MB-231 (triple-negative breast cancer cell line) cells were purchased from American Type Culture Collection (ATCC, USA). Cells were cultured in DMEM medium, supplemented with 10% (*v*/*v*) foetal bovine serum, 1% (*v*/*v*), and 100 μg/mL streptomycin and 100 U penicillin (Sigma-Aldrich, Darmstadt, Germany). HMEC-1 cells (dermal microvascular endothelial cell line) were obtained from ATCC and cultured in MCBD medium with 10% (*v*/*v*) foetal bovine serum (ATCC), 100 μg/mL streptomycin and 100 U penicillin (Sigma Aldrich, Germany), 10 ng/mL epidermal growth factor (EGF, ThermoFisher, Waltham, MA, USA), 1 µg/mL hydrocortisone and 10 mM glutamine (ATCC). Cells were grown in a humidified incubator at 37 °C with 5% (*v*/*v*) CO_2_ and 95% air. Additionally, the cell cultures were regularly tested for Mycoplasma contamination using the MycoAlert TM Mycoplasma Detection Kit (Lonza, New York, NY, USA). To keep the exponentially growing cells (with a viability higher than 90%), we evaluated their doubling time and growth rate by the trypan blue exclusion method. Thus, cells were seeded one day prior to the experiments in an accurate density and the proper volume of culture medium referred to time of incubation with tested compounds. All dilutions of investigated nanocapsules (empty or with PTX), as well as the free drug, were prepared in a culture medium, with reference to the NaCl used as a solvent in these studies.

### 2.6. MTT Assay 

The effect of empty NCs, free PTX, and PTX-loaded NCs on breast tumour cell lines and non-cancerous endothelial cells was measured by the MTT reduction assay (Sigma-Aldrich, Darmstadt, Germany). Briefly, HMEC-1, MCF-7, and MDA-MB-231 cells were seeded in 100 µL medium in 96-well transparent microtiter plates at a density of 7000 cells/well or 10,000 cells/well for breast cancer cell lines, respectively. After an overnight incubation, the cells were exposed to a range of concentrations (0–200 nM of PTX, alone or encapsulated in NCs, and 0–107 NCs/mL for empty NCs) of the investigated compounds and were then grown at 37 °C in a 5% CO_2_ atmosphere for the following time points 24, 48, 72, and 96 h. The medium containing the samples was then aspirated and MTT solution (50 mg/100 mL) was added and incubated for 3 h [28]. The dark coloured crystals were solubilized by the addition of 100 μL of DMSO. Following shaking for 30 s, the absorbance was read at 580 nm with 720 nm as the reference wavelength using a multiplate reader (Awareness Technology Inc., Palm City, FL, USA). The percentage of metabolic active cells was determined by comparing the reduction in MTT in nanocapsule-treated cells to that in the untreated control cells.

### 2.7. ATP Measurements

Viability of the cells incubated with PTX alone and PTX-NCs was determined using a CellTiter-Glo^®^ assay (Promega, Walldorf, Germany) following the manufacturer’s protocol based on the ATP luminescent measurement. HMEC-1, MCF-7, and MDA-MB-231 cells were incubated in 96-well white plates with the investigated compounds (at the PTX concentration range 0–200 nM) for 24, 48, 72, and 96 h at 37 °C. Thereafter, half of the medium was removed, replaced with 75 µL of the ATP component, and gently mixed for 5 min at room temperature [29]. Then, the luminescence was measured by a microplate luminometer (Fluoroskan Ascent FL; Labsystems, Farsta, Sweden).

### 2.8. Cell Morphology

In parallel with the cytotoxicity experiments, morphology of cells treated with tested nanosubstances was observed by phase contrast Olympus microscope (Olympus, Tokyo, Japan) equipped with a 10 × objective and a Digital Sight camera (Olympus, Tokyo, Japan).

### 2.9. CyQUANT^®^ Direct Cell Proliferation Assay

Cell proliferation was estimated by the CyQUANT^®^ assay (ThermoFisher, Waltham, MA, USA) by the intercalation of CyQUANT^®^ into DNA. The cells were treated at 37 °C for 24, 48, 72, and 96 h with free PTX or PTX-NCs (12.5, 25 and 50 nM) in a 96-well plate with an initial density of 7000–10 000 cells per well. Thereafter, the medium was removed, replaced with an equal volume of the CyQUANT component (formed by the mixture of CyQUANT^®^ direct nucleic acid stain and CyQUANT^®^ direct background suppressor I) and incubated for 60 min at 37 °C according to the manufacturer’s instructions. The fluorescence intensity was measured by a Synergy2 plate reader (BioTek, Winooski, VT, USA). In parallel, cell proliferation was visualized using the fluorogenic dye CyQUANT^®^ reagent following 72 h treatment of the cells with PTX and PTX-NCs. The fluorescent images were acquired with the Olympus microscope (Olympus, Japan) equipped with a 10× objective and a Digital Sight camera (Olympus, Japan).

### 2.10. Annexin V/PI Staining Assay

The externalisation of phosphatidylserine as the first symptom of apoptosis was determined using an Annexin V-FITC staining kit (Biovision, USA). In parallel, propidium iodide (PI) staining was conducted for cellular membrane integrity analysis. After 48 and 72 h continuous incubation with PTX alone or PTX-NCs, at doses of 12.5, 25, and 50 nM, the cells were collected by trypsinization centrifuged at 1000 rpm for 10 min, stained with the supplied fluorochromes and analysed using a LSRII BD cytometer equipped with the FACS Diva LSR II software (BD Dickinson, New York, NY, USA), the obtained data are expressed as cell percentages [30]. In parallel, the molecular features of apoptosis, after staining the cells with Annexin V and Hoechst 33,258, were visualised with a confocal microscope (Leica SP-8, Leica Microsystems, Wetzlar, Germany) with a 63×/1.40 objective (HC PL APO CS2, Leica Microsystems). Leica Application Suite X software (LAS X, Leica Microsystems, Germany) was used to obtain the images.

### 2.11. Measurement of Caspase 3/7 Activity

The activity of caspase-3/7 was assessed with luminometric assay kits (Caspase-3/7 Activity Assay, Promega, Walldorf, Germany) according to the manufacturer’s instructions. Cells were exposed to free PTX or PTX-NCs for 48 or 72 h and lysed thereafter by removing one half of the medium, replacing with an equal volume of the Caspase 3/7 assay component, and mixed for 10 min at room temperature. The intensity of luminescence was evaluated using a Fluoroskan Ascent FL plate reader (Labsystems, Stockholm, Sweden). Additionally, before the examined nanosubstances were added, the cells were pretreated for 30 min with the reversible caspase-3 inhibitor (z-VAD) at the concentration of 20 µM. The activity of caspase-3/7 was calculated as a ratio of the luminescence in the NCs-treated samples, relative to the untreated, control cells taken as 100%.

### 2.12. Mitochondrial Membrane Potential (ΔΨm)

HMEC-1, MCF-7, and MDA-MB-231 cells were seeded into 96-well microplates. After 24 h, 12.5, 25, and 50 nM of the investigated nanosubstances and PTX alone or 0.1 µM FCCP (used as a positive control) were added to the wells and the incubation was continued for up to 48 and 72 h. As we previously described, the readouts were performed on a Fluoroskan Ascent FL microplate reader (Labsystems, Stockholm, Sweden) using filter pairs of 530 nm/590 nm and 485 nm/538 nm [25]. Moreover, the ratio of JC-1 dimer to monomer in control and NCs-treated cells was visualised by fluorescence microscopy (Olympus, Japan). To avoid errors in the measurement of fluorescence intensity caused by feasible cell detachment, the obtained data were standardised to the number of viable cells estimated by the ATP measurements, following the previously described procedure. 

### 2.13. RNA Extraction and Quantitative Real-Time Polymerase Chain Reaction (qRT-PCR)

The expression levels of Apoptosis Induction Factor (AIF), Bcl-2-associated X protein (Bax), Caspase-3, and Cytochrome c (cyt c) mRNA were estimated by qRT-PCR. The primer sequences are shown in Table 1. Total RNA from cells treated with free PTX or PTX-NCs at different doses (12.5, 25 and 50 nM) for up to 48 h was extracted using TRI Reagent (Sigma Aldrich, Germany) following the manufacturer’s instructions. The concentration and purity of the RNA were measured using a NanoDrop 2000 UV–Vis Spectrophotometer (ThermoFisher Scientific/Fermentas, Vilnius, Lithuania). The relative transcription of the mRNA of apoptosis-related genes was determined by SYBR green PCR master mix (Roche, Switzerland) in a real-time PCR machine (LightCycler 480, Roche, Switzerland) [28]. The mean Ct value for duplicates was used to estimate the expression of target genes (Table 1) with standardization to the housekeeping gene used as an internal control hypoxanthine phosphoribosyltransferase 1 (HPRT1) according to the 2^−ΔCt^ formula.

### 2.14. Statistics

All measurements were obtained at least in duplicate (n ≥ 3 times). For all other studies, the calculations were performed using STATISTICA.PL software v.13 (StatSoft, Krakow, Poland). The data were expressed as mean ± SD. The normality of data distribution was conducted with the Shapiro–Wilk test whereas the homogeneity of variance was checked with the Brown–Forsythe test. Finally, the statistical analysis of the obtained results was performed using one-way ANOVA and Tukey’s multiple comparisons post-hoc test. A *p* value of 0.05 was considered significant.

## 3. Results

### 3.1. Characterization of NCs

**Figure 1:**
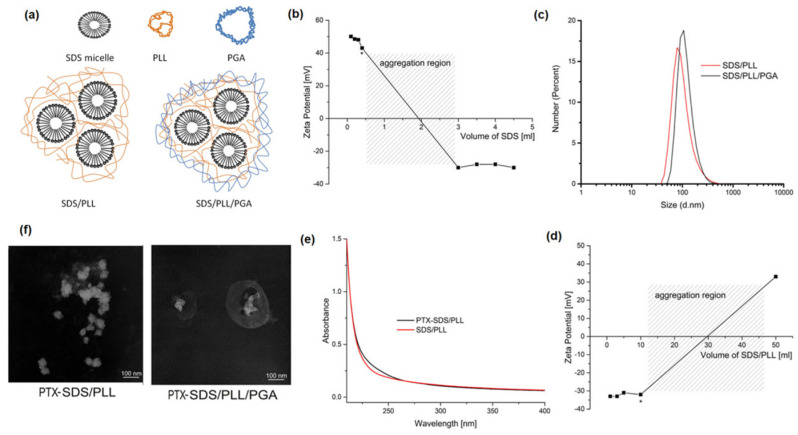
Physicochemical properties of polyelectrolyte multicore nanocarriers. (**a**) Schematic model of the SDS-based polyelectrolyte multicore nanocarriers. (**b**) Changes in the zeta potential of formed multicore nanocarriers with the addition of SDS. The optimal condition is marked by an asterisk. (**c**) The size distribution of SDS/PLL and SDS/PLL/PGA multicore nanocarriers. (**d**) Changes in the zeta potential of obtained PGA-coated nanocarriers (SDS/PLL/PGA) with the addition of SDS/PLL. The optimal condition is marked by an asterisk. (**e**) UV-Vis absorption spectra of empty multicore nanocarriers and PTX-loaded nanocarriers. (**f**) TEM images of prepared multicore nanocarriers loaded with PTX.

The micelles/polyelectrolyte complexes as multicore NCs (Figure 1a–f, Table 2) were prepared by adding the micellar solution of SDS, at the constant concentration of 10xCMC, to the PLL solution (50 ppm, 200 mL, 0.015 M NaCl) and mixing with a magnetic stirrer. To minimize the amount of free PLL as well as SDS micelles, the optimal SDS to PLL ratio was determined by monitoring changes in the zeta potential of the obtained NCs and estimating stability (Figure 1b, d). The optimal SDS volume corresponds to the point just before overcharging and the aggregation region (as marked by an asterisk in Figure 1b). At this point, most of the SDS and PLL were used to form the micelles/polycation complexes. Their zeta potential was around +43 mV, which is high enough to prevent aggregation through electrostatic stabilization. The average size determined by DLS was in the range of 70–90 nm (Figure 1c, Table 2) with a PDI < 0.25. Plasma membrane disruption is postulated to be a common property of cationic nanomaterials; therefore, positively charged NCs are not optimal for biomedical applications. Prepared SDS/PLL multicore NCs were additionally coated with PGA by the addition of SDS/PLL to the PGA solution (2000 ppm, 1 mL, 0.015 M NaCl). Again, the amount of free unabsorbed polyanion in the suspension has to be minimized; therefore, selection of the proper/optimal ratio of both components was performed by monitoring changes in the zeta potential of the obtained NCs (SDS/PLL/PGA) and examining stability. The optimal volume of SDS/PLL corresponds to the point just before overcharging and the aggregation region (as marked by an asterisk in Figure 1d). At this point, the amount of used PGA was just enough to completely coat all of the SDS/PLL present in the system; therefore, free unabsorbed polyanion in the NC suspension was minimized. The zeta potential of SDS/PLL/PGA NCs was around −32 mV, which is high enough to prevent aggregation through electrostatic stabilization. The average size was in the range of 90–110 nm (Figure 1c, Table 2), with a PDI < 0.25. One of the limitations of the transition to clinics of potential anticancer drugs is their poor solubility, ultimately resulting in poor bioavailability upon administration. Poor solubility is the result of the hydrophobicity of such drugs. Therefore, for this investigation, the model of the hydrophobic drug PTX was selected. The encapsulation of the hydrophobic components inside multicore polyelectrolyte NCs was demonstrated by the enclosure of PTX. The selected hydrophobic drug was solubilized into SDS micelles by the addition of PTX dissolved in absolute ethanol (18.3 mg/mL) to the SDS micelles suspension (10 CMC). To optimize encapsulation efficiency, the various volumes (0.1–1 mL) of PTX solution were added to SDS micelles, and the optimal (0.3 mL) was obtained after the evaporation of ethanol, and no precipitate was observed. A comparison of the UV-Vis spectra of empty and drug-loaded multicore polyelectrolyte NCs provided evidence of the successful encapsulation of PTX (Figure 1e). As PTX is practically insoluble in water, we assumed 100% encapsulation efficiency. PTX-loaded multicore NCs were additionally coated with PGA as described for empty NCs. The developed multicore polyelectrolyte NCs were visualized by TEM, which is presented in Figure 1f, and these micrographs confirmed the multicore polyelectrolyte shell structure of the NCs. Moreover, the sizes of the observed NCs were in agreement with those obtained by DLS (Figure 1c). The concentration of prepared polyelectrolyte multicore NCs determined by NTA in all NC types (empty and drug loaded SDS/PLL and SDS/PLL/PGA) was ~1 × 10^8^ nanoparticles/mL. Characterization of the prepared multicore NCs formed under optimized conditions is summarized in Table 2.

### 3.2. Cytotoxicity of SDS-Based Polyelectrolyte Nanocarriers without and with Drug

**Figure 2:**
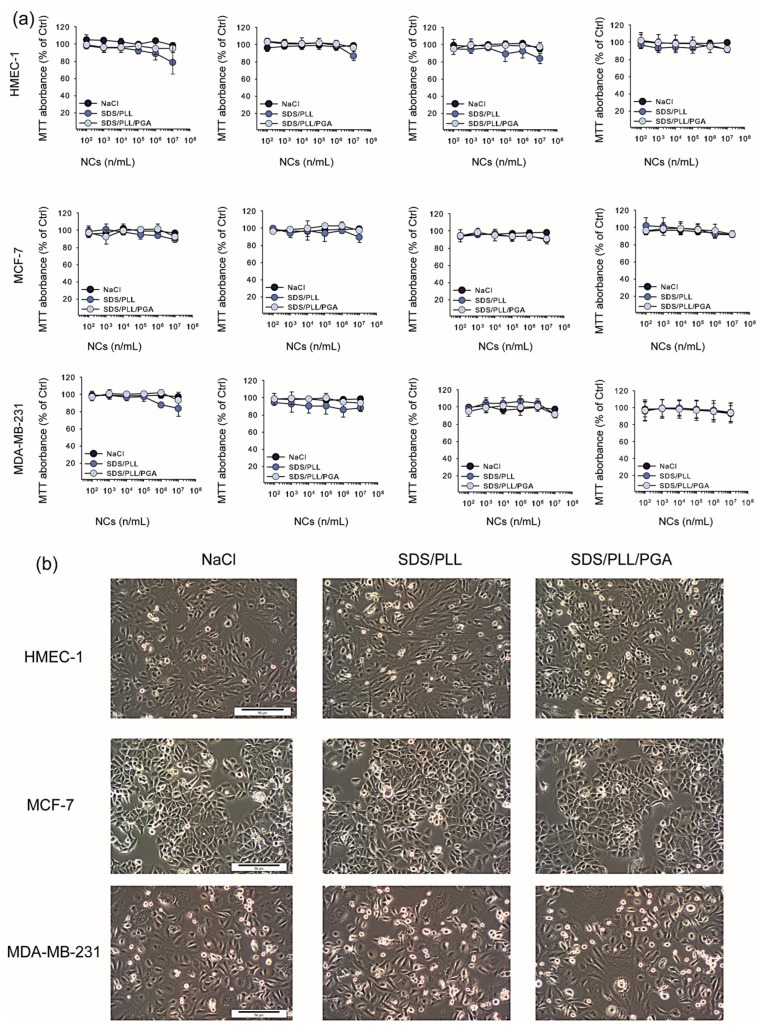
The effect of SDS/PLL and SDS/PLL/PGA on the survival of normal human breast cancer cell lines. (**a**) Cell viability estimated by the MTT assay after 24, 48, 72, and 96 h incubation with examined polyelectrolyte multicore nanocarriers. Values (calculated as percentage of untreated control cells) are mean values ± SD from three independent experiments. (**b**) Morphology of HMEC-1, MCF-7 and MDA-MB-231 cells visualized using inverted phase contrast microscopy (Olympus IX70, Japan; magnification 100) following treatment of cells for up to 96 h with SDS/PLL or SDS/PLL/PGA. The bar shown is 50 μm.

The cytotoxicity of PTX-loaded SDS/PLL and SDS/PLL/PGA NCs, empty NCs, and free PTX was analysed using the MTT and CellTiter-Glo^®^ assays following 24, 48, 72, and 96 h incubation of the nanomaterials with three different cell lines (HMEC-1, MCF-7, MDA-MB-231). The cells were exposed to the examined NCs (SDS/PLL or SDS/PLL/PGA) and, in parallel, they were treated with equivalent concentrations of PTX alone, which ranged from 3.16 nM to 200 nM. Our analysis revealed that the NCs containing PTX were much more toxic to cells than the empty NCs. The empty NCs did not significantly decrease the survival rate of all examined cell lines (Figure 2a), even at the concentration of 1 × 10^6^ NPs/mL. As shown in Figure 2b, we did not notice any morphological changes after the incubation of the cells with SDS/PLL or SDS/PLL/PGA NCs for up to 96 h. Thus, based on mitochondrial activity measurements, it was assumed that multicore polyelectrolyte NCs had no cytotoxic effect towards either normal HMEC-1 or cancerous MCF-7 and MDA-MB-231 cell lines.

**Figure 3:**
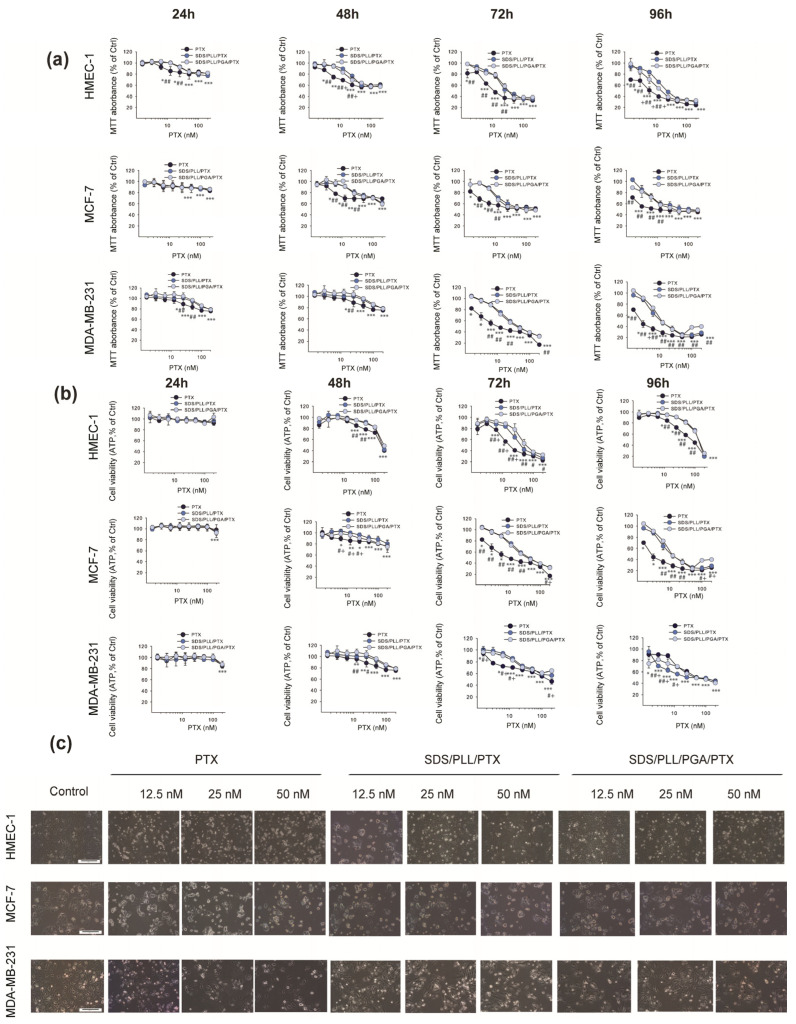
The cytotoxicity of free PTX, SDS/PLL/PTX, and SDS/PLL/PGA/PTX on HMEC-1, MCF-7, and MDA-MB-231 cells. (**a**,**b**) Viability of HMEC-1, MCF-7, and MDA-MB-231 cells was measured with the MTT assay (upper panel—(**a**)) or with CellTiter-Glo^®^ (ATP levels; lower panel—(**b**)) following incubation of cells for 24, 48, 72, and 96 h with increasing concentrations of PTX-loaded polyelectrolyte multicore nanocarriers or PTX alone. The data are shown as percentage of untreated control cells and represent mean values ± SD from three independent experiments: * *p* < 0.05, ** *p* < 0.01, *** *p* < 0.001 statistically significant changes in comparison with the untreated control cells; # *p* < 0.05, ## *p* < 0.01 significant differences between probes treated with free PTX or drug encapsulated in the examined multicore nanocarriers; + *p* < 0.05, significant differences between samples incubated with SDS/PLL/PTX or SDS/PLL/PGA/PTX (**c**) Morphological changes in HMEC-1, MCF-7, and MDA-MB-231 cells were visualized using inverted phase contrast microscopy (Olympus IX70, Japan; magnification 100) after treatment of cells for 72 h with 12.5, 25, and 50 nM of PTX encapsulated in SDS/PLL or SDS/PLL/PGA or drug alone. The bar shown is 50 μm.

The second step in the in vitro viability experiments was focused on the assessment of the cytotoxic effect of PTX encapsulated in polyelectrolyte multicore NCs. SDS/PLL/PTX as well as SDS/PLL/PGA/PTX NCs showed a similar cell-type dependent cytotoxic profile as measured by the ATP level and reduction of MTT assay (Figure 3a,b). These results demonstrated a significant increase in cytotoxicity in a dose- and time-dependent manner with the earliest toxic effect of free PTX and drug-loaded NCs after 48 h of treatment. Moreover, a comparison of data obtained from the MTT test and CellTiter-Glo^®^ assay showed a higher sensitivity of all examined cell lines if the mitochondrial redox activity of cells was estimated by the MTT assay. On the other hand, the toxicity of all PTX-loaded NCs was slightly lower in comparison to that of free PTX only if they were used at a concentration range up to 50 nM. It was observed that MDA-MB-231 cells seemed to be the most sensitive among all investigated cell lines, whereas non-cancerous HMEC-1 cells did not respond in a substantial manner, especially when treated with SDS/PLL/PTX or SDS/PLL/PGA/PTX. Interestingly, we noticed a slightly protective effect of NCs towards PTX toxicity in MCF-7 cultures. Both examined NCs loaded with the drug displayed slight differences between the type of polyelectrolyte layer that was used during synthesis, but only after 72 and 96 h of incubation with the nanosystems. These variations were considered to be due to the PTX concentrations that were chosen for further investigations.

We also visualised the morphological alterations in human breast tumour cell lines as well as non-cancerous endothelial cells incubated with PTX-loaded NCs. As shown in Figure 3c, the cells treated with SDS/PLL/PTX or SDS/PLL/PGA/PTX did not show the characteristic features of the loss of cellular homeostasis; for instance, cellular membrane destruction, cell shrinkage, and their damage. Additionally, in the presence of encapsulated PTX, the number of cells per well was markedly reduced. This indicated the cytostatic effect of the free drug and PTX-loaded SDS/PLL and SDS/PLL/PGA NCs.

### 3.3. PTX-Loaded Nanocarriers Exert Antiproliferative Action towards Breast Cancer Cell Lines and Non-Cancerous Endothelial Cells

**Figure 4:**
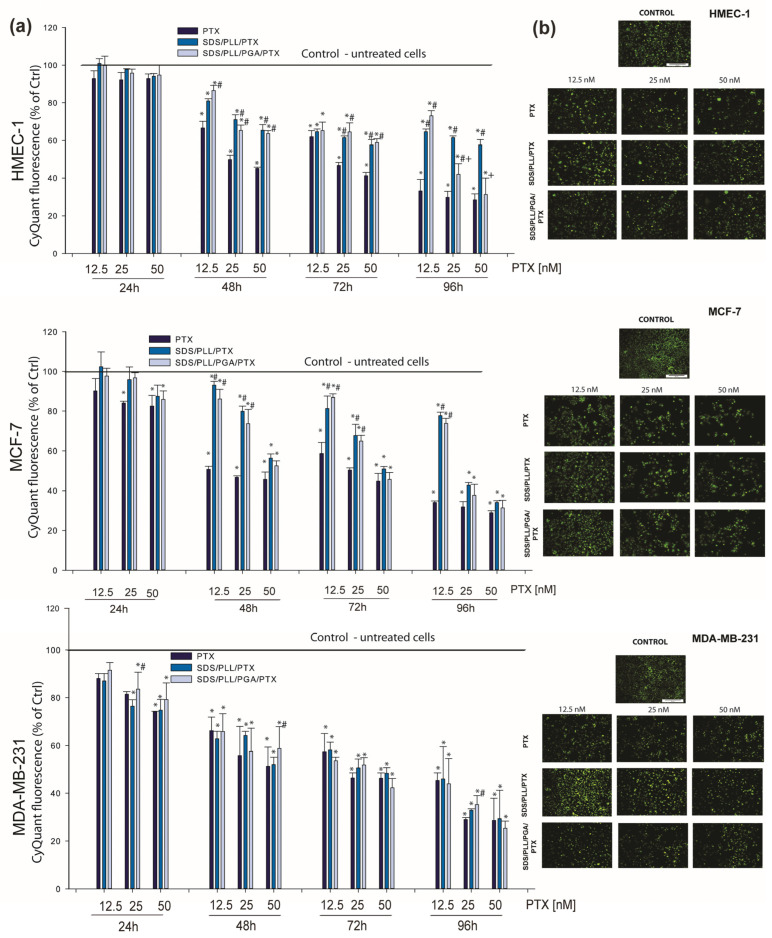
SDS/PLL/PTX and SDS/PLL/PGA/PTX reduce HMEC-1, MCF-7, and MDA-MB-231 cell proliferation. (**a**) DNA synthesis was measured in non-cancerous endothelial cells and breast tumour cell lines. The cells were treated with 12.5, 25, and 50 nM of PTX free or loaded in the investigated polyelectrolyte multicore nanocarriers for the indicated times at 37 °C in cell growth conditions. The examined cell proliferation was measured by incorporation of CyQUANT^®^ DNA-binding dye for the final 30 min of incubation with the probe. Data are shown as mean ± SD from three independent experiments: * *p* < 0.05 statistically significant changes in comparison with the untreated control cells; # *p* < 0.05 significant differences between probes treated with free PTX or drug encapsulated in the examined multicore nanocarriers; +*p* < 0.05, significant differences between samples incubated with SDS/PLL/PTX or SDS/PLL/PGA/PTX. (**b**) Visualization of the number of proliferating cells stained with CyQUANT^®^ DNA-binding dye. Images were captured at 20X (Olympus IX70, Japan; magnification 100) and the scale bar represents 50 µm.

To test whether the data obtained during the cytotoxicity experiments were associated with the inhibition of cell division, the proliferation rate of HMEC-1, MCF-7, and MDA-MB-231 cells was measured for up to 96 h. CyQuant is a cell-permeant DNA-binding dye; therefore, the DNA content was measured only in healthy cells. Based on MTT and ATP measurements, 12.5, 25, and 50 nM of PTX alone or encapsulated in polyelectrolyte NCs were selected to verify the antiproliferative properties of NC-trapped PTX. In MDA-MB-231 cancer cells, the cytostatic effect of PTX-loaded NCs was similar to that obtained with free PTX and much higher than that obtained with the empty carriers (our unpublished observations). On the other hand, we observed the same antiproliferative effect of the drug encapsulated in the investigated nanosystems in MCF-7 cells with only 50 nM of PTX. At the PTX concentrations of 25 and 50 nM, a relatively significant reduction in the cell number was observed in HMEC-1 cell cultures, especially during longer incubation times (Figure 4a). This strong growth inhibition was not noticed when normal cells were incubated with SDS/PLL/PTX and SDS/PLL/PGA/PTX NCs. For instance, 12.5 nM of the free drug diminished DNA synthesis by more than 60%, whereas SDS/PLL/PTX and SDS/PLL/PGA/PTX triggered only a decrease of 21% and 17% in HMEC-1 cell proliferation, respectively. Our results on cell growth reduction were also confirmed by microscopic observation (Figure 4b). A significant decline in the cell number with up to 72 h of incubation with PTX-loaded multicore polyelectrolyte NCs was observed.

### 3.4. PTX Trapped in Polyelectrolyte Multicore Nanocarriers Possess Pro-Apoptotic Activity

**Figure 5:**
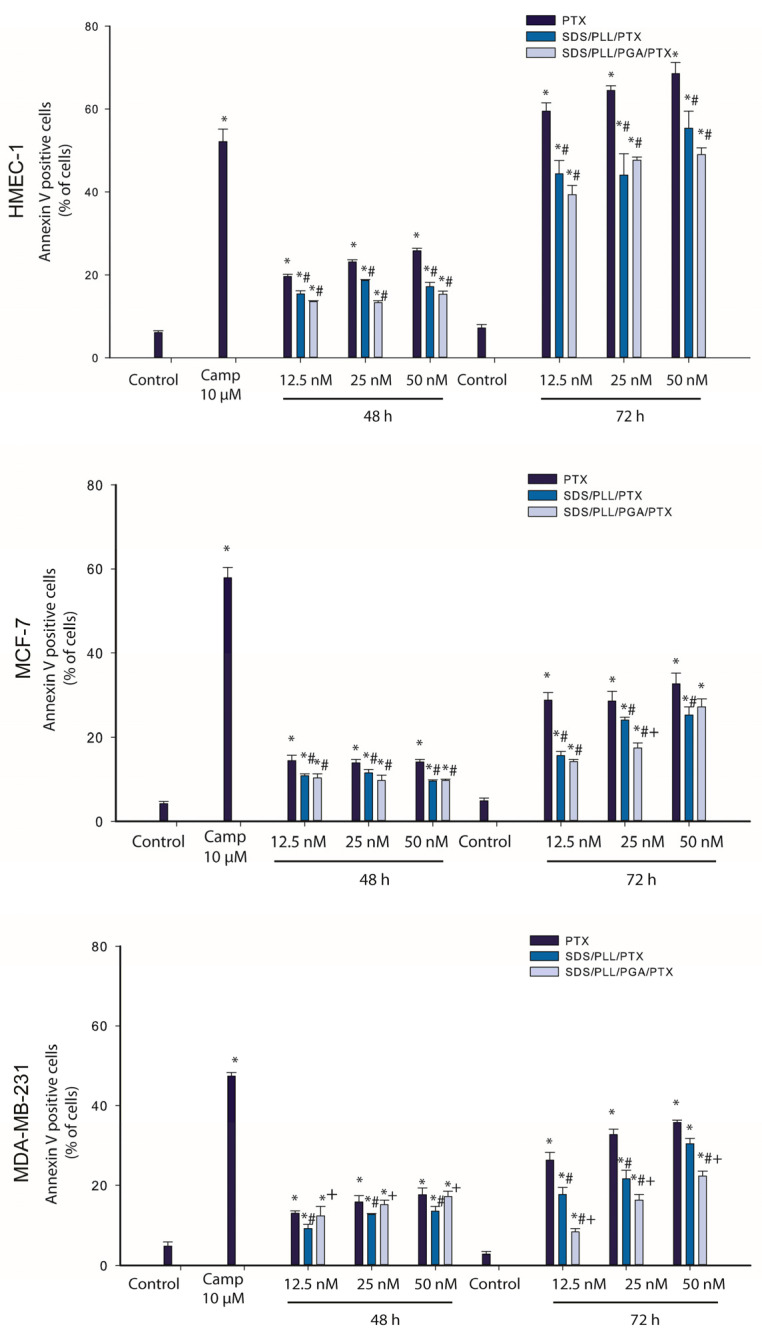
Externalisation of phosphatidylserine in HMEC-1, MCF-7, and MDA-MB-231 cell lines treated with PTX-loaded nanocarriers. Breast cancer cell lines and non-cancerous endothelial cells were incubated with 12.5, 25, and 50 nM of SDS/PLL/PTX, SDS/PLL/PGA/PTX, or PTX alone for 48 and 72 h. Camptothecin (10 µM) was used as a positive control. The figure shows the mean percentages (±SD) of Annexin-V binding cells. Additionally, the integrity of cellular membrane was determined by propidium iodide staining and for control cells, the percentage of Annexin-V binding cells and PI positive cells did not exceed 3%; * *p* < 0.05 statistically significant changes in comparison with the untreated control cells; # *p* < 0.05 significant differences between probes treated with free PTX or drug encapsulated in the examined multicore nanocarriers; + *p* < 0.05, significant differences between samples incubated with SDS/PLL/PTX or SDS/PLL/PGA/PTX.

An observed reduction in the proliferation rate in cell culture is often an attribute of modified cellular homeostasis when programmed cell death mechanisms are activated. In accordance with the statement that PTX is a well-known anticancer agent that induces apoptosis [31], we determined whether PTX-loaded NCs triggered a transfer of phosphatidylserine to the external leaflet of cell membranes. As a positive control, treatment with 10 µM Camptothecin for 24 h was performed, which has previously been described as a common apoptosis inducer [32] Thus, upon incubation with 10 µM of Camptothecin, a profound growth of Annexin-V binding cells was observed, which was reflected by phosphatidylserine externalisation. As shown in Figure 5, treatment with free PTX, SDS/PLL/PTX, or SDS/PLL/PTX induced a gradual growth in the percentage of Annexin-V binding in all examined cell lines. 

**Figure 6:**
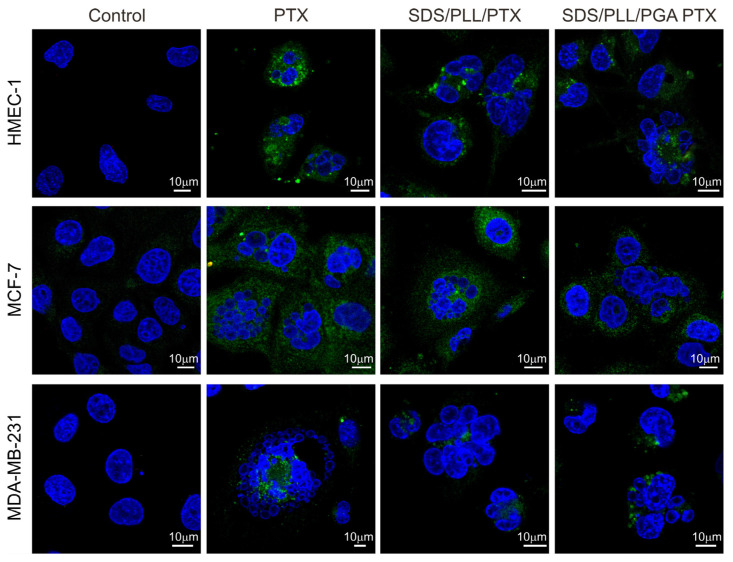
Apoptotic cell death was assessed in examined cancer and non-cancer cells treated with the indicated concentrations of PTX alone, SDS/PLL/PTX, or SDS/PLL/PGA/PTX for up to 72 h using the Annexin V assay kit. In addition, nuclei were counterstained with Hoechst 33258 followed by fluorescence microscopy analysis. Note the typical morphological features of apoptosis: loss of the structural framework of the nuclei, condensation of chromatin, cell shrinkage, nuclear fragmentation, and detachment of apoptotic bodies. Scale bar 10 µm. The cells were analysed under a confocal fluorescence microscope (Leica microsystems, Germany).

In the case of MCF-7 and MDA-MB-231 cells 48 h after incubation with SDS/PLL/PTX, SDS/PLL/PGA/PTX, or PTX alone, the percentage of Annexin-V binding cells reached approximately 12%, 11%, and 13%, respectively. When incubation was prolonged for up to 72 h, it was revealed that the externalisation of phosphatidylserine was approximately two-fold higher (around 35%) in both breast cancer cell lines exposed to all forms of PTX. In non-cancerous HMEC-1 cells, a maximal growth in the number of Annexin V binding cells was also observed after 72 h exposure to free PTX (66.5%), SDS/PLL/PTX (54.3%), and SDS/PLL/PGA/PTX (48.2%). In parallel, PI staining was performed to assess cell membrane integrity and the number of PI positive cells did not exceed 10% in both normal and cancer cells. In all cells exposed to SDS/PLL/PTX or SDS/PLL/PGA/PTX, a progressive increase in the morphological hallmarks accompanying programmed cell death was observed. In addition to flow cytometry measurements, cells were also stained with Hoechst 33258 (Figure 6). Apparent cellular shrinkage, chromatin condensation and marginalization, and the formation of apoptotic bodies and their fragmentation were seen after 48 h incubation with PTX encapsulated in SDS/PLL or SDS/PLL/PGA NCs or free PTX. 

**Figure 7:**
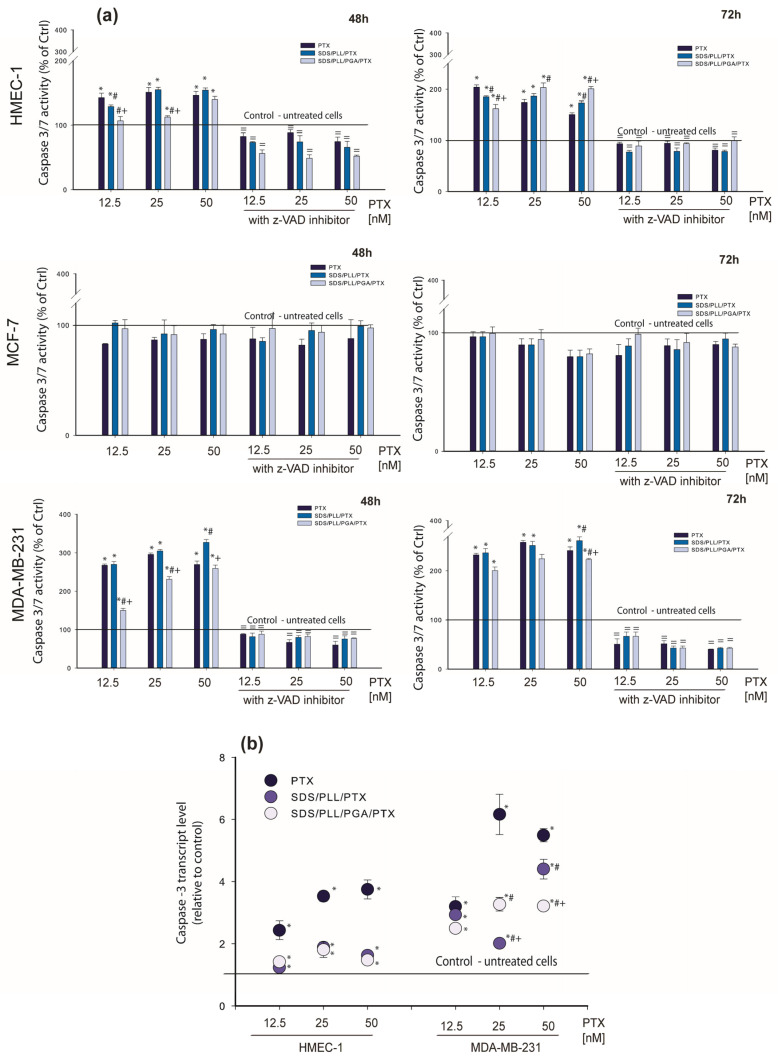
Caspase-3/7 is involved in apoptosis induction in PTX-trapped polyelectrolyte multicore nanocarriers. (**a**) Time-dependent changes in caspase-3/7 activity in HMEC-1 and MDA-MB-231 cells treated with various concentrations of PTX-loaded nanocarriers (12.5; 25 and 50 nM) for 24, 48, and 72 h. (**b**) Caspase-3 transcription level (relative to HPRT1) in the cell line exposed to PTX, SDS/PLL/PTX, and SDS/PLL/PGA/PTX for 24 h. Results of caspase-3/7 activity and caspase-3 mRNA expression are the means ± SD of three independent experiments; * *p* < 0.05 statistically significant changes in comparison with the untreated control cells; # *p* < 0.05 significant differences between probes treated with free PTX or drug encapsulated in the examined multicore nanocarriers; + *p* < 0.05, significant differences between samples incubated with SDS/PLL/PTX or SDS/PLL/PGA/PTX, = *p* < 0.05, significant differences between samples preincubated with caspase-3 inhibitor (z-VAD).

Keeping in mind that the observed executive stage of apoptosis requires the activation of many enzymes, the recruitment of caspase-3 or -7 after cell treatment with PTX-loaded NCs was assessed. Estimations of the protein level, changes in the enzyme activity, as well as the transcriptional level by the measurement of the caspase 3′ mRNA copies were performed. The results presented in Figure 7a indicate that SDS/PLL/PTX as well as SDS/PLL/PGA/PTX activated caspase-3 both in MDA-MB-231 cancer and HMEC-1 normal cells. MCF-7 cells do not express caspase-3 [33].

Maximal increase in caspase-3 activity was noted after the incubation of HMEC-1 cells for up to 72 h with 12.5 nM of PTX (204%), 25 nM of SDS/PLL/PTX (186.5%), and 50 nM of SDS/PLL/PGA/PTX (203%). In the MDA-MB-231 cell line, the highest growth in caspase-3 activity was noted when cells were incubated with 50 nM of PTX encapsulated in SDS/PLL NCs (328%) for up to 48 h. The preincubation of cells with a caspase-3 inhibitor (z-VAD) confirmed that the alterations in the enzyme activity were a consequence of programmed cell death induction. On the other hand, a slightly earlier increase (48 h of treatment with the examined compounds) in caspase-3′ transcription was observed in HMEC-1 cells and MDA-MB-231 cells. As shown in Figure 7b, caspase-3′ mRNA level reached statistically significant values when non-cancer endothelial cells and the MDA-MB-231 cell line were treated with all forms of PTX. 

### 3.5. Mitochondrial Homeostasis Is Altered by PTX-Loaded Multicore Polyelectrolyte Nanocarriers

**Figure 8:**
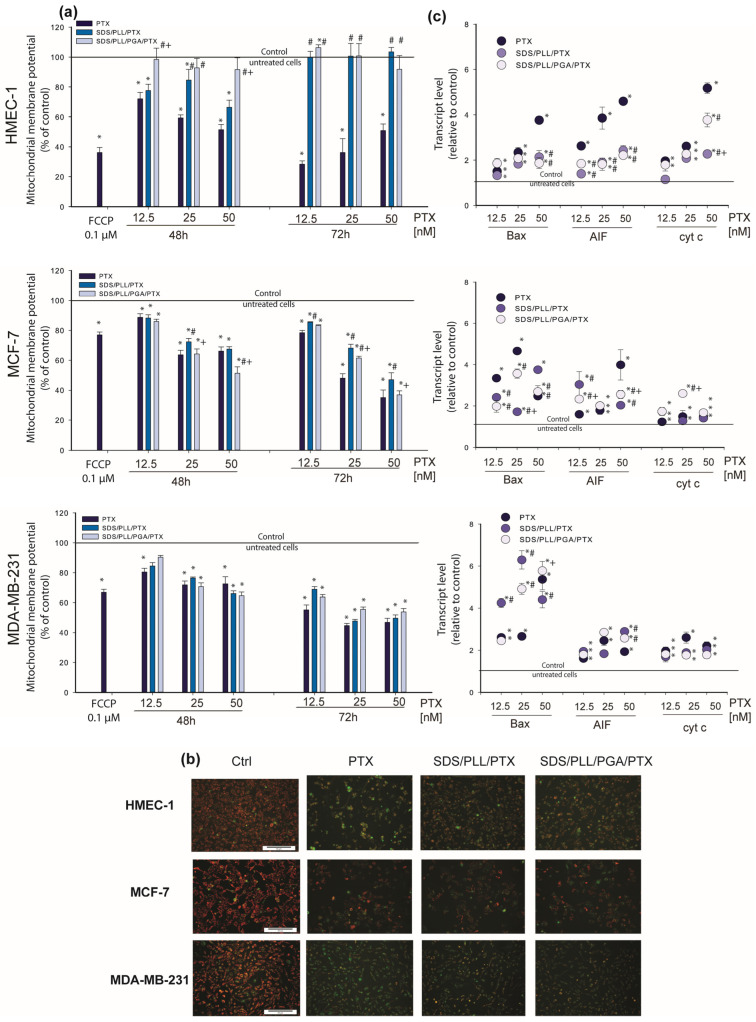
Mitochondrial stress triggered by polyelectrolyte multicore nanocarriers with encapsulated PTX in HMEC-1 and MDA-MB-231 cells. (**a**) Changes in mitochondrial membrane potential of non-cancerous endothelial cells and breast cancer cell lines incubated with the indicated concentrations of PTX, SDS/PLL/PTX, or SDS/PLL/PGA/PTX for up to 48 and 72 h; FCCP (0.01 µM) was used as a positive control. Fluorescence ratio of JC-1 dimers to JC-1 monomers in the control was assumed to be 100%. Results are presented as means ± SD of three experiments. * *p* < 0.05 statistically significant changes in comparison with the untreated control cells; # *p* < 0.05 significant differences between probes treated with free PTX or drug encapsulated in the examined multicore nanocarriers; + *p* < 0.05, significant differences between samples incubated with SDS/PLL/PTX or SDS/PLL/PGA/PTX. (**b**) Fluorescent microscopy images of untreated control cells and cells treated with PTX alone or loaded in SDS/PLL or SDS/PLL/PGA for 72 h at 37◦C. Red fluorescence of JC-1 dimers is present in the cell areas with high mitochondrial membrane potential, while green fluorescence of JC-1 monomers is common in the cell areas with low mitochondrial membrane potential. The JC-1-stained cells were visualized with a fluorescence microscope (Olympus IX70, Japan), 10× 10, scale bar 50 μm. (**c**) Bax, *AIF*, and *Cytochrome c* genes transcript expression (relative to HPRT1 housekeeping gene) in HMEC-1, MCF-7, and MDA-MB-231 cells exposed to the examined nanosubstances loaded with PTX or the free drug. Data are shown as means ± SD of three experiments in duplicate. * *p* < 0.05 statistically significant changes in comparison with the untreated control cells; # *p* < 0.05 significant differences between probes treated with free PTX or drug encapsulated in the examined multicore nanocarriers; + *p* < 0.05, significant differences between samples incubated with SDS/PLL/PTX or SDS/PLL/PGA/PTX.

An overlap between the increase in caspase-3/7 activity and the increase in the transcription of caspase-3′ mRNA was noted. Afterwards, PTX disturbances in mitochondrial homeostasis triggered by the NCs were assessed. It has been reported that caspase-3 interacts with caspase-9, another protease enzyme involved in the release of cytochrome c from mitochondria and apoptosome formation [34]. To assess whether PTX-loaded NCs act as a mitochondria-dependent cell death stimulus, firstly, alterations in mitochondrial membrane potential were measured. Preincubation with 0.1 µM of FCCP applied as a positive control [35] caused a profound decrease in Δψm. With regard to the incubation of HMEC-1 cells with PTX-loaded polyelectrolyte multicore NCs, it was found that SDS/PLL/PTX as well as SDS/PLL/PGA/PTX cause a significant decrease in Δψm (around 66%) when treated for 48 h and 72 h. In contrast, the incubation of HMEC-1 cells with 25 nM of free PTX triggered dose-dependent changes in Δψm (Figure 8a) which decreased by 36% after 72 h incubation. MCF-7 and MDA-MB-231 cell cultures showed comparable effects in both types of PTX-loaded NCs at 50 nM that diminished the mitochondrial membrane potential to nearly 50%. The data obtained during Δψm measurements were visualised as well by fluorescence microscopy observations. As shown in Figure 8b, PTX-loaded multicore NCs triggered a notable increase in the green fluorescence of JC-1 monomers in both breast tumour cell lines, which confirmed a reduction in Δψm. These differences were similar when cells were treated with PTX alone. As incubation with SDS/PLL/PTX or SDS/PLL/PGA/PTX potentially affects mitochondrial function, alterations in the transcription of key mitochondrial and apoptotic-dependent genes by the polyelectrolyte multicore NCs were also investigated. Interestingly, MDA-MB-231 cells incubated with 50 nM of SDS/PLL/PTX or SDS/PLL/PGA/PTX elevated the level of cyt c mRNA, resulting in up to 2.6-fold and 2.2-fold increases, respectively (Figure 8c). Furthermore, the incubation of MCF-7 cells with 12.5 nM and 25 nM of PTX-loaded SDS/PLL/PGA NCs increased cyt c mRNA that corresponded well with the increase in Bax and AIF transcription of 5.3-fold and 4.2-fold, respectively. However, in non-cancer cells, free PTX triggered a more prominent increase in transcription of the investigated genes, whereas the changes induced by SDS/PLL/PTX or SDS/PLL/PGA/PTX were not so extensive.

## 4. Discussion

Although the development of nanotaxanes has recently been observed, the clinical application of PTX-based nanoformulations is significantly limited. The unsatisfactory drug release acquired resistance of cancer tissue and the off-target distribution of PTX loaded nanosystems are the main factors that decrease their therapeutic efficacy. For instance, Nanoxel^®^ is incapable of selectively targeting and releasing the encapsulated PTX in tumour cells. Apart from Nanoxel^®^, other nanotaxanes such as Cynviloq^®^, Bevetex^®^, and Taxoprexin^®^ have shown similar unsatisfactory results [19,36]. In our previous work, the application of the LbL technique for the possible synthesis of various nanocapsule cores containing nanoemulsion droplets was described [37]. During the manufacturing of such nanoemulsion cores, oil-soluble surfactants were used which, together with the oppositely charged polyelectrolyte, form an interfacial complex stabilizing the nanoemulsion droplet. This droplet, together with polyelectrolyte coatings formed by the LbL technique, creates a single core nanocapsule varying in size from 70 to 200 nm [38]. Here, the use of SDS as a water-soluble surfactant which with the oppositely charged polyelectrolyte can form a volume complex (nanomicelles/polyelectrolyte) was proposed. This complex forms the multicore polyelectrolyte NC, with the correct internal conditions for the encapsulation of hydrophobic drugs, e.g., taxanes. Therefore, in this study, newly invented polyelectrolyte multicore NCs consisting of micelles in water-soluble SDS with a solubilized lipophilic active agent PTX were developed. These biocompatible NCs were coated with an oppositely charged pair of polyelectrolytes, PLL and PGA, respectively. The SDS-based NCs, synthesised according to the LbL technique, were optimized for use as a DDS (Figure 1f). In addition, the active ingredient, in this case the hydrophobic anticancer drug PTX, was relatively easy to incorporate into the NC structure. Thus, we presumed that SDS/PLL as well as SDS/PLL/PGA nanosystems could actively deliver PTX directly to the tumour niche, mostly due to their bioavailability and the EPR effect in cancer tissue.

With regard to the biological studies, PTX encapsulated in SDS-based polyelectrolyte multicore NCs reduced cancer cell division and induced the programmed cell death pathway. SDS/PLL/PTX as well as SDS/PLL/PGA/PTX elevated caspase-3 enzymatic activity and upregulated caspase-3 mRNA expression. Incubation with the drug-loaded nanosystems enhanced Bax, AIF, and cytochrome-c mRNA levels and triggered a decrease in (ΔΨm). Following the alteration in the transcription of mitochondrial genes, the mitochondrial redox activity of the cells (estimated by the MTT assay) was markedly reduced, indicating that PTX released from SDS/PLL or SDS/PLL/PGA nanosystems disturbed cellular homeostasis. Moreover, cell proliferation and DNA synthesis were abolished when cancer cells were incubated with PTX-loaded SDS-based polyelectrolyte multicore NCs. Surprisingly, empty NCs (without PTX) had a neutral effect on cell viability. Notably, neither SDS/PLL nor SDS/PLL/PGA appeared to induce cellular dysfunctions, as indicated by the lack of morphological changes. Finally, when non-cancer HMEC-1 cells were treated with free PTX, the toxic effect of the drug was stronger in comparison to that triggered by PTX encapsulated in the polyelectrolyte multicore nanosystems. A similar effect was noted in MCF-7 cells. These various cellular responses confirmed the release of PTX from synthesized NCs; however, release kinetics could not be determined due to the system’s complexity in which the drug is released and the inability to mimic such conditions during the experiments.

Substantial research has documented that PTX can act solely as a suppressor of the microtubule polymerization and consequently inhibits cancer cell proliferation [39]. It was proved that the toxicity of PTX is schedule-dependent in breast and lung tumour cells [40]. For instance, Giannakakou et al. [41] demonstrated that a decrease in the proliferation of lung A549 as well as breast MCF-7 cells was triggered by PTX at concentrations above 12 nM. Here, in this article, DNA synthesis was measured as the marker of cell division rate. Interestingly, a dose-dependent effect of all examined forms of PTX in the triple negative MDA-MB-231 cell line was found. This was not observed in the MCF-7 cell line, which revealed the substantial resistance of cells to 12.5 nM of SDS/PLL/PTX and SDS/PLL/PGA/PTX NCs. The difference in the cellular response of both breast cancer cell lines can be explained by the expression of HER2 receptors on MCF-7 cell lines, which are mostly treated with aromatase inhibitors (Trozole) and Tamoxifen, an antagonist of ER [42]. Even though MCF-7 and MDA-MB-231 cells have high demand for iron ions, these two cell lines express CD71 protein (the receptor for iron transport) in a different way. Corte-Rodríguez et al. showed a four-fold increase in the CD71 protein expression of triple negative breast cancer cultures in comparison to MCF-7 cells [43]. Finally, it is well known that various cell lines may have different contributions from the various endocytic uptake mechanisms (e.g., clathrin mediated, clathrin-independent uptake (including caveolae) and macropinocytosis) [44,45], and thus this issue will be continued in our further studies.

Several reports have documented that the lower percentage of cell proliferation after PTX treatment directly refers to the fraction of metabolically active cells. Thus, the intracellular ATP level was quantified in normal and cancer cells incubated with PTX alone and PTX-loaded NCs. A statistically significant difference between the tested forms of PTX was noted in MCF-7 cells and HMEC-1 cells. Our data confirmed that when incubation with both encapsulated forms of PTX was prolonged for up to 96 h, the normal endothelial cell line did not reveal a substantial sensitivity towards either SDS/PLL/PTX or SDS/PLL/PGA/PTX. On the other hand, this observation was confirmed by the MTT assay only for the lower concentrations of the examined compounds. If breast cancer cells were exposed to at least 50 nM of free PTX or drug encapsulated in tested nanosystems, a similar cytotoxic effect was observed (Figure 3a,b). It is in fact quite common to see that drug-loaded NCs and the free drug give a similar toxic effect on cells in vitro [46]. Such studies demonstrate that the drug is entering the cells and delivering its effect to the intracellular target within the time frame of the incubation period. The main advantage of incorporating drugs in nanocapsules is to change the biodistribution and pharmacokinetics of the drugs, as demonstrated more than 20 years ago for Doxil^®^/Caelyx^®^, i.e., the first NP-based therapeutic product to enter the market [47,48].

Our results verify how important it is to perform different viability assays when studying the cytotoxic effect of nanomaterials. It was recently mentioned that a specific regulatory course of treatment based on novel nanodevices can provide robust scientific insights supported by the theranostic properties of NPs [49]. This issue is extremely crucial regarding the validation of the toxicity of pure nanomaterials that seem to be good candidates for drug delivery systems. With regard to bare nanomaterial cytotoxicity, the concentration, size, surface area, and charge and PDI index must be taken into consideration during analysis of their toxic effect [50]. Moreover, the binding of NPs to the cellular membrane and their cellular uptake may activate multidimensional cellular stress responses [51]. In NP-mediated drug delivery, when stress responses induced by a nonsubstance sensitize the target cells to an intended drug effect, the influence of the NCs itself may be extremely valuable [52]. Consequently, the role of nanomaterials cannot be limited only to passive drug carriers, but due to their properties, the effect of NPs may be complementary with the encapsulated drug’s toxicity [53]. In our work, the MTT assay and microscopic observations were performed to determine whether empty NCs were toxic to normal and cancer cell lines. In this context, the present work demonstrated that SDS-based polyelectrolyte multicore NCs were not toxic and had a neutral effect on HMEC-1, MCF-7, and MDA-MB-231 cell cultures. This was also observed by Karabasz et al. When AOT-based polyelectrolyte NCs were tested in the CT26-CEA mouse colon adenocarcinoma cell line, it was shown that cell viability was strictly dependent on the NCs charge, and the survival rate of CT26-CEA cells was decreased by positively, but not by negatively, charged NCs [37]. The reduction in breast cancer cell viability after their treatment with SDS/PLL/PTX and SDS/PLL/PGA/PTX was mostly observed after prolonged incubation for up to 72 or 96 h. This observation could even be directly linked with the aim of the multicore nanocapsules application and their ability to localise within the tumour tissue. Consequently, it suggests that our nanoobjects may facilitate a sustained drug release and in turn will provide overall lower off-target toxicity and increased therapeutic efficacy.

Keeping in mind that MTT reduction to formazan takes place in mitochondria, our next step was to measure the alteration of the mitochondrial membrane potential in cells treated with PTX alone or PTX encapsulated in SDS/PLL/PGA NCs. A dose-dependent irreversible effect of PTX-loaded NCs was noticed only in MCF-7 and MDA-MB-231 cells, whereas in the normal HMEC-1 cell line, a marked drop in Δψm was observed mainly after free PTX treatment. The alterations in the mitochondrial response of cancer and normal cells towards the tested formulation of PTX agreed with the expression of genes crucial for maintaining mitochondrial homeostasis. We discovered that the alterations in Bax, AIF, and cytochrome c mRNA levels were responsible for mitochondrial apoptosis. Similar observations were obtained by Ren et al., who noted that in canine mammary gland tumour cells, PTX induced the overexpression of the pro-apoptotic protein Bax [54]. These changes were responsible for triggering mitochondria-dependent apoptosis through the disruption of Δψm and the consequent release of cytochrome c from mitochondria into the cytoplasm and cleavage of the caspase-3 protein [55]. 

Our data confirmed that free PTX and PTX-loaded NCs stimulated in HMEC-1 and MDA-MB-231 cell lines a growth in caspase-3/7 enzymatic activity as well as the overexpression of casp-3′ mRNA. Along with these changes, we proved the alteration of phosphatidylserine externalisation, the other hallmark of apoptosis. As described above, incubation with all tested concentrations of PTX alone or PTX trapped in polyelectrolyte multicore NCs exerted similar potential and enhanced the number of Annexin V positive cells up to 69%. The same phenomenon was noticed in the A2780 ovarian cancer cell line treated with the liposomal PTX nanosystem (L-PTX) [56]. In line with the flow cytometry measurements, we observed cellular shrinkage and chromatin condensation and formation (as well fragmentation) of membrane-bound apoptotic bodies in a dose-dependent manner when cells were treated for up to 72 h with the examined compounds. Our data (Figure 7) support those by Tran et al. [57], who showed collapsed or disassembled nuclei in MCF-7, BT-474, and MDA-MB-231 cells treated with PTX-ART-loaded PLGA NPs and subsequently stained with Hoechst 33258. 

## 5. Conclusions

**Figure 9:**
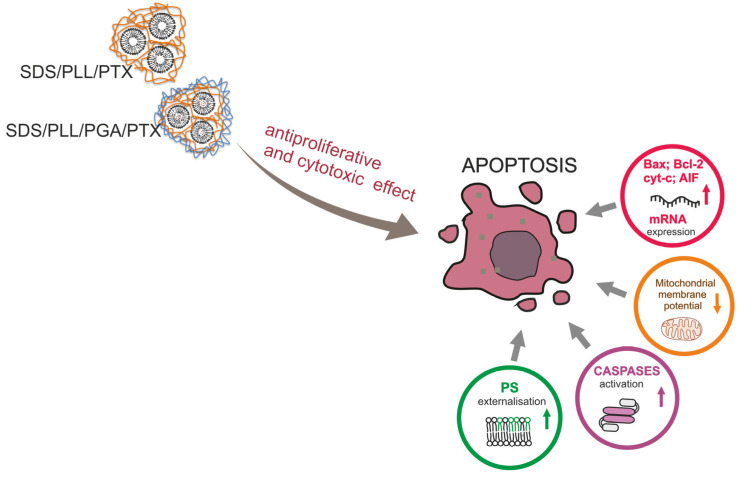
Model of SDS-based loaded with PTX polyelectrolyte multicore nanocarriers indicating our key findings. Two different breast cancer cellular models (MCF-7 and MDA-MB-231 cells) were proposed in this study. In parallel, experiments were performed on a non-cancer HMEC-1 cell line. We proved that toxicity index of SDS/PLL/PTX and SDS/PLL/PGA/PTX nanocapsules increased with a prolonged time of treatment and had a similar cytotoxicity in comparison with free PTX. Our data confirmed a decrease in the degree of proliferation and reduction in cell number, mainly after 72 and 96 h of incubation with the encapsulated drug. During microscopic observations, it was noted that significant proapoptotic cell membrane damage occurred after incubation of cells with examined compounds, which was confirmed by the phosphatidylserine externalization and caspase3/7 assays. In addition, it was shown that SDS/PLL/PTX and SDS/PLL/PGA/PTX induced mitochondrial dysfunction that altered an expression of important genes for apoptosis (*Bax, cyt c, and AIF*). In summary, we established SDS-based multicore polyelectrolyte nanocapsules as a tool for highly hydrophobic drug delivery systems in chemotherapy.

In conclusion, our data demonstrate that PTX encapsulated in SDS/PLL/PTX and SDS/PLL/PGA/PTX NCs displays cytotoxic properties and suppresses the proliferation of cancer cells through the induction of programmed cell death (Figure 9). It was identified that NCs loaded with PTX effectively kill cancer cells, especially when incubation with NCs was prolonged for up to 72 h. Moreover, the high sensitivity of triple negative cancer cells towards PTX encapsulated in the SDS-based polyelectrolyte nanocarriers showed examined nanocapsules as good candidates for further biomedical studies. 

## 6. Patents

Patent claim no. WIPO ST 10/C PL443843.

## Figures and Tables

**Table 1:** Primer sequences used for RT-PCR.

Gene	Strand	Sequence 5′-> 3′
Hypoxanthine-guanine phosphoribosyltransferase (HPRT1)	Forward Reverse	TGACACTGGCAAAACAATGCA GGTCCTTTTCACCAGCAAGCT
Bcl2-like protein 4 (Bax)	Forward Reverse	GTTTCATCCAGGATCGAGCAG CATCTTCTTCCAGATGGTGA
Apoptosis induction factor (AIF)	Forward Reverse	AGACGATCCCAAATAATGCAG TAGCTCTAGGTGATCTTGG
Caspase -3 (casp-3)	Forward Reverse	AGGCCCCTGGATACTCTTACACAG TCAGTGTATCCTCTCCCCAGATG
Cytochrome c (Cyt c)	Forward Reverse	AGGCCCCTGGATACTCTTACACAG TCAGTGTATCCTCTCCCCAGATG

**Table 2:** Characterization of the multicore polyelectrolyte nanocarriers.

Abbreviation	The Range of Size	Zeta Potential	Nanocarrier Concentration	PTX Concentration
SDS/PLL	70–90 nm	+43 mV	1 × 10^8^ nanoparticles/mL	-
SDS/PLL/PGA	90–110 nm	−33 mV	1 × 10^8^ nanoparticles/mL	-
SDS/PLL/PTX	70–90 nm	+49 mV	1 × 10^8^ nanoparticles/mL	2.07 mg/L
SDS/PLL/PGA/PTX	90–10 nm	−32 mV	1 × 10^8^ nanoparticles/mL	1.85 mg/L

## Data Availability

The datasets presented during the current study are available from the corresponding author on reasonable request.
